# LncRNA GAS5 suppresses TGF-β1-induced transformation of pulmonary pericytes into myofibroblasts by recruiting KDM5B and promoting H3K4me2/3 demethylation of the PDGFRα/β promoter

**DOI:** 10.1186/s10020-023-00620-x

**Published:** 2023-03-14

**Authors:** Yichun Wang, Diyu Chen, Han Xie, Shuhua Zhou, Mingwang Jia, Xiaobo He, Feifei Guo, Yihuan Lai, Xiao Xiao Tang

**Affiliations:** 1 Department of Critical Care Medicine, Key Laboratory for Major Obstetric Diseases of Guangdong Province, The Third Affiliated Hospital of Guangzhou Medical University, No. 63 Duobao Road, Liwan District, Guangzhou, 510150 Guangdong Province People’s Republic of China; 2 CAS Key Laboratory of Regenerative Biology and Guangdong Provincial Key Laboratory of Stem Cell and Regenerative Medicine, Guangzhou Institutes of Biomedicine and Health, Chinese Academy of Sciences, Guangzhou, 510150 Guangdong Province People’s Republic of China; 3 Department of Obstetrics and Gynecology, Key Laboratory for Major Obstetric Diseases of Guangdong Province, Key Laboratory of Reproduction and Genetics of Guangdong Higher Education Institutes, The Third Affiliated Hospital of Guangzhou Medical University, Guangzhou, People’s Republic of China; 4 State Key Laboratory of Respiratory Disease, National Clinical Research Center for Respiratory Disease, National Center for Respiratory Medicine, Guangzhou Institute of Respiratory Health, The First Affiliated Hospital of Guangzhou Medical University, No. 195 Dongfeng West Road, Yuexiu District, Guangzhou 510150 Guangdong Province, People’s Republic of China

**Keywords:** GAS5, Idiopathic pulmonary fibrosis, PDGFRα/β, Myofibroblasts

## Abstract

**Background:**

Idiopathic pulmonary fibrosis (IPF) is a condition that may cause persistent pulmonary damage. The transformation of pericytes into myofibroblasts has been recognized as a key player during IPF progression. This study aimed to investigate the functions of lncRNA growth arrest-specific transcript 5 (GAS5) in myofibroblast transformation during IPF progression.

**Methods:**

We created a mouse model of pulmonary fibrosis (PF) via intratracheal administration of bleomycin. Pericytes were challenged with exogenous transforming growth factor-β1 (TGF-β1). To determine the expression of target molecules, we employed quantitative reverse transcription-polymerase chain reaction, Western blotting, and immunohistochemical and immunofluorescence staining. The pathological changes in the lungs were evaluated via H&E and Masson staining. Furthermore, the subcellular distribution of GAS5 was examined using FISH. Dual-luciferase reporter assay, ChIP, RNA pull-down, and RIP experiments were conducted to determine the molecular interaction.

**Results:**

GAS5 expression decreased whereas PDGFRα/β expression increased in the lungs of IPF patients and mice with bleomycin-induced PF. The in vitro overexpression of GAS5 or silencing of PDGFRα/β inhibited the TGF-β1-induced differentiation of pericytes to myofibroblasts, as evidenced by the upregulation of pericyte markers NG2 and desmin as well as downregulation of myofibroblast markers α-SMA and collagen I. Further mechanistic analysis revealed that GAS5 recruited KDM5B to promote H3K4me2/3 demethylation, thereby suppressing PDGFRα/β expression. In addition, KDM5B overexpression inhibited pericyte–myofibroblast transformation and counteracted the promotional effect of GAS5 knockdown on pericyte–myofibroblast transformation. Lung fibrosis in mice was attenuated by GAS5 overexpression but promoted by GAS5 deficiency.

**Conclusion:**

GAS5 represses pericyte–myofibroblast transformation by inhibiting PDGFRα/β expression via KDM5B-mediated H3K4me2/3 demethylation in IPF, identifying GAS5 as an intervention target for IPF.

**Supplementary Information:**

The online version contains supplementary material available at 10.1186/s10020-023-00620-x.

## Introduction

Idiopathic pulmonary fibrosis (IPF) is an irreversible and fatal lung disease featured by progressive scarring of the pulmonary parenchyma with a median survival of 3 years. It currently affects 14–43 per 100,000 individuals, and its incidence increases with age (Maher et al. 2021; Raghu et al. 2006; Yu and Tang 2022). According to current pathogenic theories, fibroblast activation and differentiation play important roles in IPF (Yamaguchi et al. 2020). The exposure of quiescent fibroblasts to a variety of profibrotic mediators, such as transforming growth factor-beta 1 (TGF-β1) and platelet-derived growth factor (PDGF), results in phenotypic differentiation into myofibroblasts, which causes excess deposition of extracellular matrix (ECM) during IPF progression (Yin et al. 2018; Xie et al. 2020). Over the past several years, researchers have found that pericytes are a substantial source of myofibroblasts and that atypical pericyte activation may be a significant cause of IPF (Chou et al. 2020). The biological functions of PDGF are mediated by binding to specific receptors (PDGFRα/β) (Chou et al. 2020; Djudjaj and Boor 2019). Blockage of the PDGFRα/β signaling pathway activation can delay IPF progression (Kishi et al. 2018; Vuorinen et al. 2007; Wang et al. 2021). Thus, elucidation of the regulatory mechanisms of the PDGFRα/β signaling pathway is crucial for IPF prevention and treatment.

Lysine-specific demethylase 5B (KDM5B), a demethylase at the K4 position of H3, acts as a transcriptional inhibitor in various biological processes. For instance, KDM5B contributed to colorectal cancer proliferation via CDX2 inhibition by H3K4me3 demethylation (Huang et al. 2020). It has been suggested that HIF-1α can trigger tissue fibrosis through KDM5B-mediated transcriptional repression via H3K4me2/3 demethylation (Salminen et al. 2016). To date, it remains unclear whether KDM5B is involved in the pathogenesis of IPF. As predicted by the AnimalTFDB and RPISeq databases, KDM5B could bind to the PDGFRα/β promoter. Thus, we speculated that KDM5B transcriptionally represses PDGFRα/β via H3K4me2/3 demethylation to delay IPF progression.

Long noncoding RNAs (lncRNAs) are specialized RNAs that have been proposed as therapeutic targets for a variety of diseases, including IPF. Chen et al. reported that lncRNA CTD-2528L19.6 attenuated IPF via fibroblast activation repression (Chen et al. 2021). It has been recognized that lncRNA growth arrest-specific 5 (GAS5) participates in multiple lung disorders, such as asthma and lung cancer (Poulet et al. 2020). However, the involvement of GAS5 in the pathological development of IPF remains unclear. In our preliminary experiments, we found that GAS5 was downregulated in patients and mice with IPF. Furthermore, bioinformatics analysis revealed that GAS5 could directly bind to KDM5B. In this context, we speculated that GAS5 recruited KDM5B to restrain PDGFRα/β transcription by promoting H3K4me2/3 demethylation, thereby inhibiting the transformation of lung pericytes to myofibroblasts.

This study aimed to validate the aforementioned hypothesis in TGF-β1-exposed lung pericytes and bleomycin-treated mice. Our findings clarify the regulatory role of GAS5 in IPF progression, identifying GAS5 as a novel intervention target for IPF therapy.

## Materials and methods

### Clinical samples

Lung tissues were obtained from 33 IPF patients (male, 25; female, 8; mean age, 40.14 ± 13.63 years) who underwent surgical resection at the third Affiliated Hospital of Guangzhou Medical University. Prior to the procedure, none of the patients had received any therapy. In addition, normal lung tissues (tumor-adjacent tissues) were obtained and used as controls. Informed consent was obtained from all participants.

### Isolation and culture of primary pericytes

All experiments have been approved by the ethics committee of the Third Affiliated Hospital of Guangzhou Medical University, and pericytes were purified as previously described (Hung et al. 2017). Fresh lung tissues were collected from mice and cut into small pieces. After digestion and filtration through nylon meshes, single cells were resuspended in culture media. Pericytes with negative CD45, CD31, and CD326 expressions and positive PDGFRβ expression were selected using MACS (Miltenyi Biotec, USA). The isolated pericytes were seeded into gelatin-coated culture plates and maintained using DMEM/F-12 (11330032, Thermo Fisher Scientific Inc., Waltham, MA, USA) supplemented with 10% FBS (10100, Thermo Fisher).

### Cell transfection

For the induction of fibrogenesis, mouse pulmonary microvascular pericytes were treated with TGF-β1 (HY-P7117, MCE, NJ, USA) for 30 min at 37 °C. The shRNA targeting GAS5 (shGAS5), shPDGFRα, shPDGFRβ, shKDM5B, and negative control (shNC) were synthesized by GenePharma (Shanghai, China). The specific sequences for shRNAs are presented in Table 1. For the overexpression of GAS5 and PDGFRα/β, GAS5 and PDGFRα/β sequences amplified by RT-qPCR were ligated with the pcDNA3.0 plasmids (#13031, Addgene, Watertown, MA, USA) to establish the pcDNA3.0-GAS5 and pcDNA3.0-PDGFRα/β recombinant plasmids, respectively. The primers used for establishing the overexpression constructs are presented in Table 2. The aforementioned plasmids and sequences were transfected into cells using Lipofectamine 3000 (L3000001, Thermo Fisher Scientific) following the manufacturer’s protocols.

**Table 1:** Sequences for shRNAs

shGAS5#1 targeting sequence	5′-GGATCTCACAGCCAGTTCTGT-3′
shGAS5#2 targeting sequence	5′-GGATCTCACAGCCAGTTCTGT-3′
shGAS5#3 targeting sequence	5′-GCAATGTGCTAGAATAGAAGA-3′
shGAS5#4 targeting sequence	5′-GAGGCTGGATAGACAGTTTGA-3′
shKDM5B#1 targeting sequence	5′-CGGTGCTATTTCTATTCCTTA-3′
shKDM5B#2 targeting sequence	5′-GCCTACATCATGTGAAAGAAT-3′
shKDM5B#3 targeting sequence	5′-CCTGAAATTCAGGAGCTTTAT-3′
shKDM5B#4 targeting sequence	5′-ATCGCTTGCTGCACCGTTATT-3′
shPDGFRα#1 targeting sequence	5′-GCCAGCTCTTATTACCCTCTA-3′
shPDGFRα#2 targeting sequence	5′-GCCACATTTGAACATTGTGAA-3′
shPDGFRα#3 targeting sequence	5′-CCTGGAGAAGTGAGAAACAAA-3′
shPDGFRα#4 targeting sequence	5′-GATGATCTGCAAGCATATTAA-3′
shPDGFRβ#1 targeting sequence	5′-CCATGCTTAATGAATGCTGTT-3′
shPDGFRβ#2 targeting sequence	5′-CCTTGAATGAAGTCAACACTT-3′
shPDGFRβ#3 targeting sequence	5′-GAAGCGGGCTACTATACTATG-3′
shPDGFRβ#4 targeting sequence	5′-GATGTCACTGAGACGACAATT-3′

**Table 2:** The primers used for establishing the overexpression constructs

GAS5 (gene ID: 14455)	F: 5′-CTTTCGGAGCTGTGCGGCATTCTGA-3′ R: 5′-TTCATGTTATAATACACTTTAATGG-3′
PDGFRα (gene ID: 18595)	F: 5′-ATGAGGACCTGGGCTTGCCTGCTGC-3′ R: 5′-TTAGGTGGGTTTTAACCTTTTCCTT-3′
PDGFRβ (gene ID: 18596)	F: 5′-ATGCTGAGCGACCACTCCATCCGCT-3′ R: 5′-CTAGGCTCCGAGGGTCTCCTTCAGG-3′
KDM5B (gene ID: 75605)	F: 5′-ATGGAGCCGGCCACCACGCTGCCCC-3′ R: 5′-TTACTTTCGGCTTGGTGCGTCCTTC-3′

### Mouse model of pulmonary fibrosis (PF)

As previously described (Pan et al. 2020), 7–8-week-old male C57BL/6 mice weighing 20–22 g were obtained from Hunan Slac Jingda Laboratory Animal Co., Ltd. (Changsha, China). The mice were randomly assigned into six groups (n = 7 per group): control, bleomycin, bleomycin + NC, bleomycin + GAS5, bleomycin + shNC, and bleomycin + shGAS5. Intraperitoneal injection with 30-mg/kg sodium pentobarbital was adopted to anesthetize the mice. To inducePF, 2.5-mg/kg bleomycin (HY-108345, MCE) was intratracheally administered to the mice. The control group was administered with the same volume of PBS (10010001, Thermo Fisher Scientific). Five days before bleomycin administration, the mice were intratracheally injected with lentiviruses containing overexpression of GAS5, shGAS5, or NC (1 × 10^7^ TU, GenePharma). Then, 2 weeks after bleomycin administration, the mice were sacrificed, and lung tissues were collected for further analysis. All animal protocols were approved by the Animal Care and Use Committee of Guangzhou Medical University.

### Hematoxylin and eosin staining

Before being paraffin-embedded, the lung samples of mice were fixed in 4% paraformaldehyde (P1110, Solarbio, Beijing, China). Subsequently, the lungs were cut into 4-µm slices and stained with the H&E staining kit (G1120, Solarbio) and examined under a microscope (Olympus, Japan) for pathological and fibrosis assessment.

### Masson staining

Collagen deposition was evaluated via Masson staining on lung slices. In brief, the 4-μm slices of lungs were stained using the Masson trichrome staining kit (G1340, Solarbio). The images were photographed under a microscope.

### Immunohistochemical staining

The 4-µm paraffin-embedded lung slices were dewaxed and rehydrated in ethanol gradient. After blocking endogenous peroxidases with 0.3% hydrogen peroxide (7722-84-1, Sigma–Aldrich, Saint Louis, MO, USA), the slices were probed overnight at 4 °C with primary antibodies against α-SMA (ab124964, 1:50, Abcam, Cambridge, MA, UK), collagen I (ab88147, 1:50, Abcam), and desmin (ab227651, 1:2000, Abcam). Then, the slices were rinsed with PBS and reacted with the secondary antibody. Subsequently, 3,3′-diaminobenzidine was used to visualize the slices. A light microscope was used to obtain the photographs.

### Real-time quantitative polymerase chain reaction (RT-qPCR)

Using the TRIzol reagent (T9424, Sigma–Aldrich), total RNA was isolated from the pericytes and tissues, followed by reverse transcription into cDNA using the PrimeScript RT reagent kit (RR047AA, Takara, Tokyo, Japan). Then, qPCR was performed using the SYBR Premix Ex Taq II kit (RR820A, Takara). GAPDH was used as the housekeeping gene to calculate the relative gene expression using the 2^−ΔΔCT^ method as previously described (Zuo et al. 2022). The primers used in RT-qPCR are listed in Table 3.

**Table 3:** Primers used for RT-qPCR analysis

Genes	Primer sequences (5′–3′)
Human GAS5	F: 5′-GTGAGGTATGGTGCTGGGTG-3′
	R: 5′-GCCAATGGCTTGAGTTAGGC-3′
Human PDGFRA	F: 5′-GAAGCTGTCAACCTGCATGA-3′
	R: 5′-CTTCCTTAGCACGGATCAGC-3′
Human PDGFRB	F: 5′-CCCAATGAGGGTGACAACGA-3′
	R: 5′-AAGCTATCCTCTGCTTCCGC-3′
Human GAPDH	F: 5′-CCAGGTGGTCTCCTCTGA-3′
	R: 5′-GCTGTAGCCAAATCGTTGT-3′
Mouse Gas5	F: 5′-GAATGGCAGTGTGGACCTCT-3′
	R: 5′-CAGCCTCAAACTCCACCATT-3′
Mouse Pdgfra	F: 5′-TGGCATGATGGTCGATTCTA-3′
	R: 5′-CGCTGAGGTGGTAGAAGGAG-3′
Mouse Pdgfrb	F: 5′-CCTTCTCCAGTGTGCTGACA-3′
	R: 5′-TCATGTAGCGTCACCTCCAG-3′
Mouse Gapdh	F: 5′-AGCCCAAGATGCCCTTCAGT-3′
	R: 5′-CCGTGTTCCTACCCCCAATG-3′

### Western blotting

The cells or tissues were lysed in RIPA solution (P0013B, Beyotime, Haimen, China) and quantified using BCA protein assay (P0012, Beyotime). The protein (50 µg) was separated on SDS-PAGE and then transferred to PVDF membranes (3010040001, Roche, Basel, Switzerland). After blocking with 5% skimmed milk for 1 h, the membranes were incubated with primary antibodies overnight at 4 °C. The following primary antibodies were used: PDGFR-α (ab203491, 1:1000, Abcam), PDGFR-β (ab69506, 1:1000, Abcam), α-SMA (ab124964, 1:2000, Abcam), NG-2 (ab275024, 1:1000, Abcam), collagen I (ab260043, 1:1000, Abcam), desmin (ab227651, 1:5000, Abcam), KDM5B (ab181089, 1:1000, Abcam), H3K4me2 (#9725, 1:1000, CST, USA), H3K4me3 (#9751, 1:1000, CST, Danvers, MA, USA), and β-actin (ab8226, 1:1000, Abcam). After incubation with a secondary antibody, the membranes were treated with the ECL-chemiluminescent kit (34580, Thermo Fisher Scientific) to visualize the protein bands.

### Immunofluorescence staining

Primary pericytes were fixed in 4% paraformaldehyde, permeabilized with 0.1% Triton X-100 (T8787, Sigma–Aldrich), and blocked with 1% BSA (A1595, Sigma–Aldrich). The antibodies, including α-SMA (ab124964, 1:200, Abcam) and desmin (ab227651, 1:500, Abcam), were applied overnight at 4 °C. The pericytes were probed with goat anti-rabbit IgG (H + L) secondary antibody (ab150077, 1:200, Abcam) for 1 h. A fluorescence microscope (Zeiss, Germany) was used to observe and photograph the outcomes of the immunofluorescence assays.

### Fluorescence in situ hybridization (FISH)

FISH was employed to determine GAS5 localization in pericytes. Briefly, the pericytes were seeded in a 24-well plate. After fixation and permeabilization, the cells were probed with GAS5 probe. Subsequently, the cells were rinsed in a hybridization solution and stained with DAPI (MBD0015, Sigma–Aldrich) in the dark. Images were obtained using a laser scanning confocal microscope (Leica, Germany).

### Chromatin immunoprecipitation (ChIP) assay

ChIP assay was conducted using the Magna ChIP and EZ-Magna ChIP kit (17-10461, Millipore, Billerica, MA, USA). Sequences for the PDGFRα/β promoter containing the wild-type (WT) or mutant (MUT) binding sites in KDM5B are presented in Table 4. Generally, pericytes were extracted and cross-linked with 1% formaldehyde before being treated with 125-mM glycine (67419, Sigma–Aldrich). The cell suspension was then sonicated and precipitated with anti-KDM5B (#15327, CST), anti-H3K4me2 (#9725, CST), anti-H3K4me3 (#9751, CST), or anti-control rabbit IgG (ab37415, Abcam). PDGFRα/β enrichment in the immunoprecipitated complex was assessed via quantitative PCR.

**Table 4:** Sequences for PDGFRα/β promoter containing the wild type (MT) or mutant (MUT) binding sites in KDM5B

WT-PDGFRα-BS1(-582/-558)	5′-GGGAGAGAAACAAACGGAGGAGCTG-3′
WT-PDGFRα-BS2(-487/-463)	5′-GAAGGAGAAGGTAAGGGAGAGGAAA-3′
WT-PDGFRα-BS3(-487/-463)	5′-AAAAAAAAAGAAAAGAAAAAGAAAA-3′
MUT-PDGFRα-BS1(-582/-558)	5′- CCCTCTCTTTGTTTGCCTCCTCGAC-3′
MUT-PDGFRα-BS2(-487/-463)	5′-CTTCCTCTTCCATTCCCTCTCCTTT-3′
MUT-PDGFRα-BS3(-487/-463)	5′-TTTTTTTTCTTTTCTTTTTCTTTT-3′
WT-PDGFRβ-BS1(-1432/-1417)	5′-CTCTTCCTGTTTCCTC-3′
WT-PDGFRβ-BS2(-196/-172)	5′-GGAAAGGAGGAAGAAAAACAAGAAA-3′
WT-PDGFRβ-BS3(-165/-141)	5′-GGAAAAGAAAGAGAGGAAAAAAAA-3′
MUT-PDGFRβ-BS1(-1432/-1417)	5′-GAGAAGGACAAAGGAG-3′
MUT-PDGFRβ-BS2(-196/-172)	5′-CCTTTCCTCCTTCTTTTTGTTCTTT-3′
MUT-PDGFRβ-BS3(-165/-141)	5′-CCTTTTCTTTCTCTCCTTTTTTTT-3′

### Dual-luciferase reporter assay

A luciferase reporter assay was conducted to confirm the target relationship between KDM5B and PDGFRα/β. The sequences of the PDGFRα/β promoter containing the WT or MUT binding sites in KDM5B were synthesized and introduced into the pmirGLO vector (E1330, Promega, Madison, WI, USA). In 96-well plates, 293 T cells were co-transfected with the aforementioned reporter vectors and shKDM5B or shNC using Lipofectamine 3000 (L3000001, Thermo Fisher Scientific). After 48 h, the luciferase activity was measured using a dual-luciferase reporter system (E1960, Promega).

### RNA pull-down assay

The biotin-labeled GAS5 probe was provided by RiBobio (Guangzhou, China) and transfected into pericytes. Then, cell lysate was prepared using lysis buffer and incubated with streptavidin–agarose beads (16-126, Millipore) for 3 h at 4 °C. The RNA-binding protein complexes in agarose beads were washed and boiled at 95 °C–100 °C. Finally, the eluted protein was assessed via Western blotting.

### RNA immunoprecipitation assay

Pericytes were lysed using the lysis buffer from the Magna RIP kit (17-700, Millipore). The cell lysate was treated overnight at 4 °C with RIP buffer containing magnetic beads pre-coated with anti-IgG (#3900, CST) or anti-KDM5B (#15327, CST). To remove the protein, proteinase K (70663, Sigma–Aldrich) was added to the eluted samples at 55 °C for 30 min. Subsequently, the immunoprecipitated RNA was isolated, and GAS5 enrichment was determined via RT-qPCR.

### Statistical analysis

The data are expressed as mean ± standard deviation (SD). Statistical analysis was conducted using GraphPad Prism. Student’s *t*-test was employed to determine the difference between two groups. One-way analysis of variance was employed to compare multiple groups, followed by Tukey’s post hoc test. *P* < 0.05 was considered to indicate statistical significance.

## Results

### Downregulation of GAS5 and upregulation of PDGFR α/β in the lung of IPF patients and mice with bleomycin-induced pulmonary fibrosis

**Fig. 1:**
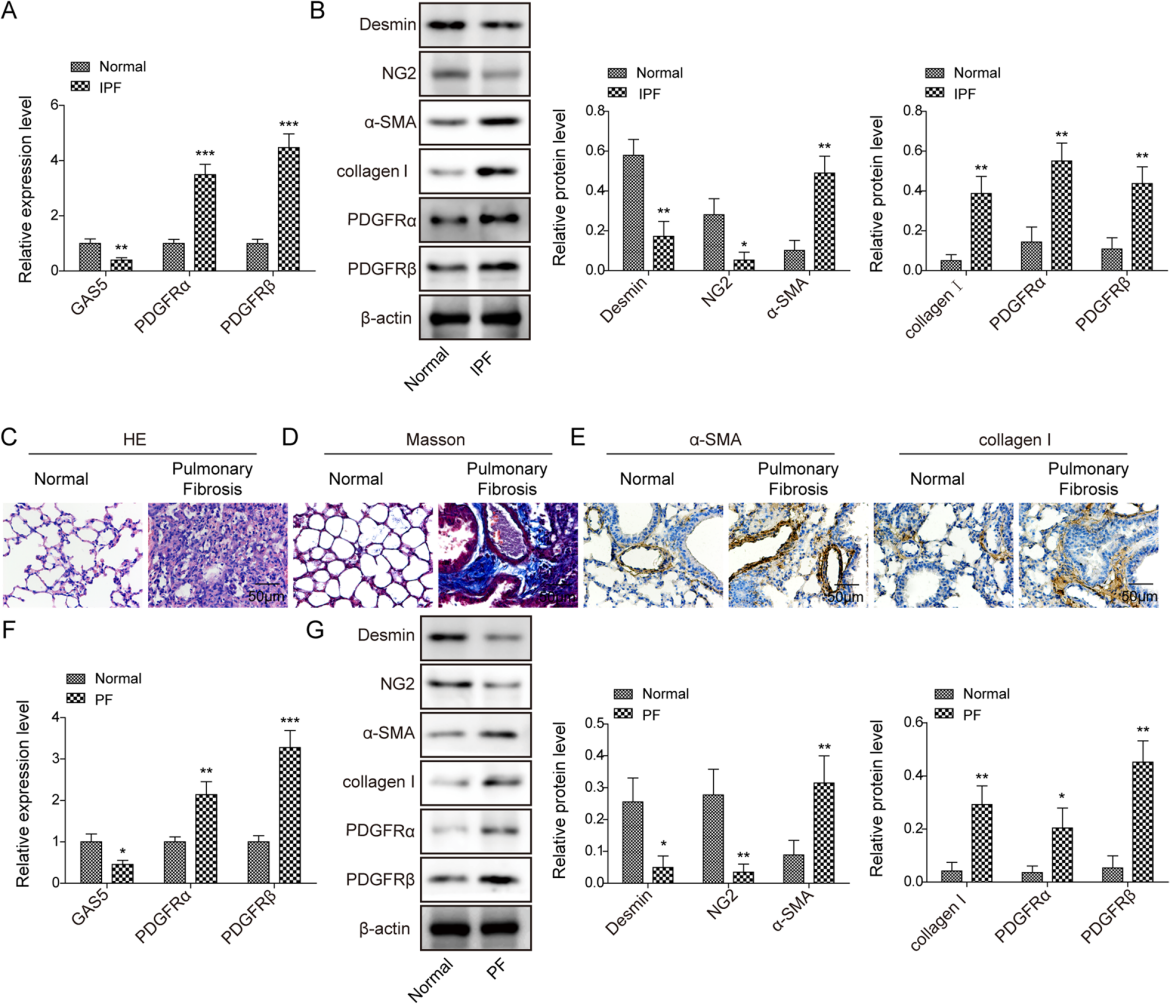
A low GAS5 expression and a high PDGFR α/β expression were observed in the lungs of patients and mice with IPF. **A** The expressions of GAS5 and PDGFR α/β in the IPF specimens were examined via RT-qPCR. **B** Western blotting was employed to explore the expressions of desmin, NG2, α-SMA, collagen I, and PDGFR α/β in IPF patients. **C** The pathological changes in the lung tissues of bleomycin-induced mice were evaluated via H&E staining. **D** Masson staining was used to determine collagen formation in the lung tissues. **E** Immunohistochemical staining was employed to detect the expressions of α-SMA and collagen I. **F** RT-qPCR analysis of the expressions of GAS5 and PDGFR α/β in IPF mice. **G** Western blotting was used to examine the expressions of desmin, NG2, α-SMA, collagen I, and PDGFR α/β in the lungs. For A and B, n = 33; for C–F, n = 6. *P < 0.05, **P < 0.01, ***P < 0.001

First, the dysregulation of GAS5 and PDGFR α/β in IPF was evaluated via RT-qPCR. We found that the GAS5 expression decreased whereas the PDGFR α/β expression increased in the IPF lung tissues (Fig. 1A). Furthermore, the protein levels of PDGFR α/β, collagen I, and α-SMA were enhanced, but the levels of pericyte markers desmin and NG2 decreased in the IPF specimens (Fig. 1B). To further confirm the above findings, a bleomycin-induced mouse model of PF was created. The fibrosis and collagen deposition in the lung tissues of bleomycin-challenged mice were observed via H&E and Masson staining (Fig. 1C, D). Immunohistochemical staining further confirmed the enhanced expressions of α-SMA and collagen I in PF mice (Fig. 1E). Similarly, GAS5, desmin, and NG2 were downregulated whereas PDGFR α/β, collagen I, and α-SMA were upregulated in the bleomycin-induced PF mouse model (Fig. 1F, G). Taken together, GAS5 was aberrantly downregulated and PDGFR α/β was upregulated in patients and mice with IPF.

### TGF-β1 exposure-induced phenotypic differentiation of pericytes into myofibroblasts in a dose- and time-dependent manner

**Fig. 2:**
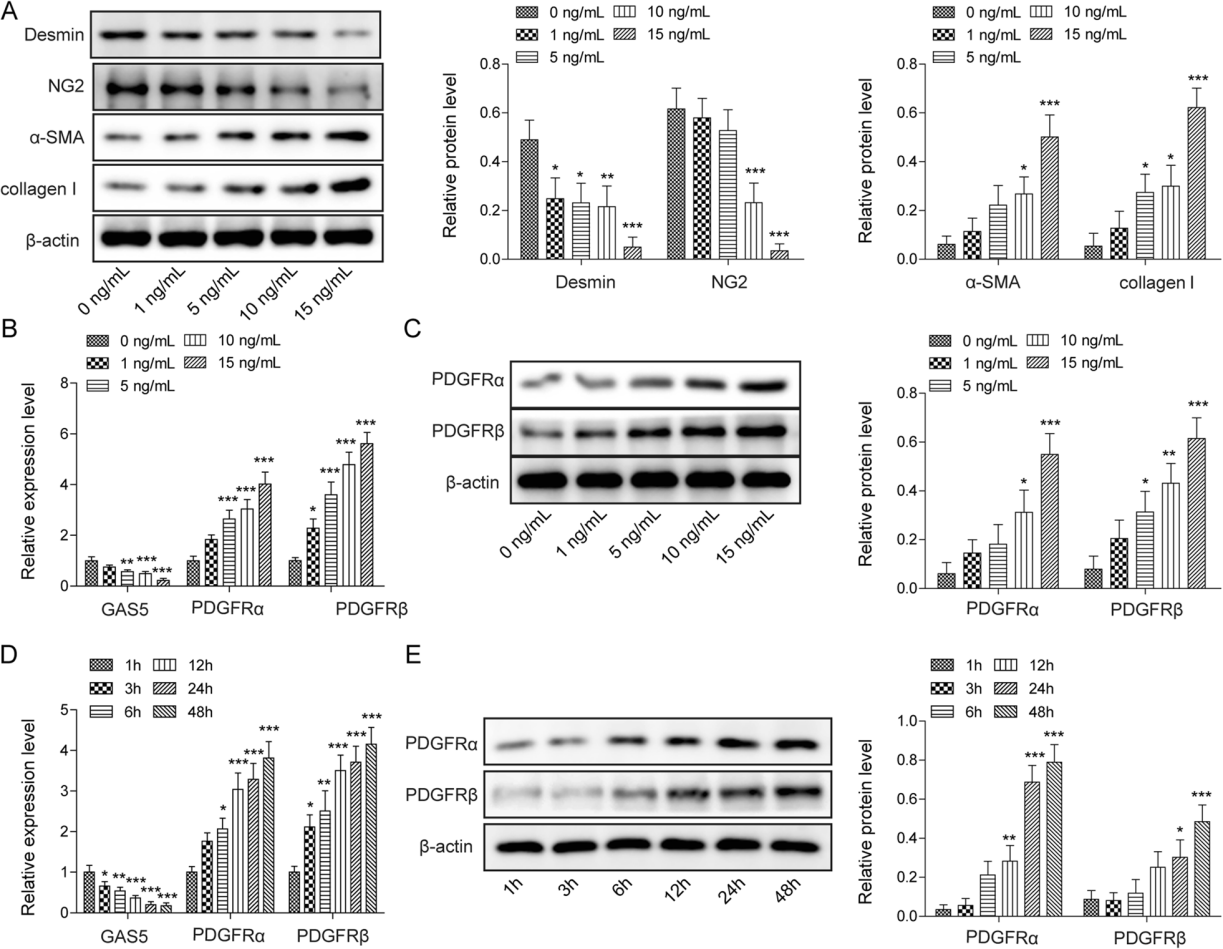
TGF-β1 induced pericyte–myofibroblast transformation in a dose- or time-dependent manner. Pericytes were exposed to different TGF-β1 concentrations (0, 1, 5, 10, and 15 ng/mL) for 12 h. **A** Western blotting analysis of the levels of pericyte markers (desmin, NG-2) and myofibroblast markers (α-SMA, collagen I). **B** RT-qPCR analysis of the expressions of GAS5 and PDGFR α/β in pericytes. **C** The expression of PDGFR α/β was evaluated via Western blotting. Pericytes were treated with 10 ng/mL of TGF-β1 at different time periods (1, 3, 6, 12, 24, and 48 h). **D** The expressions of GAS5 and PDGFR α/β were examined via RT-qPCR. **E** Western blotting analysis of the expression of PDGFR α/β. For A–E, n = 3. *P < 0.05, **P < 0.01, ***P < 0.001

We tested the effect of TGF-β1 on the transformation of pericytes into myofibroblasts. The results of Western blotting indicated that the levels of myofibroblasts (collagen I and α-SMA) were dose-dependently elevated whereas the levels of pericyte markers (desmin and NG2) were declined by TGF-β1 stimulation (Fig. 2A). Furthermore, TGF-β1 exposure reduced GAS5 expression and increased PDGFR α/β expression dose-dependently (Fig. 2B, C). It was also found that with the extension of time, GAS5 was downregulated and PDGFR α/β were upregulated in TGF-β1-exposed pericytes (Fig. 2D, E). These data suggested that TGF-β1 induced the phenotypic transformation of pericytes into myofibroblasts in a dose- and time-dependent manner.

### GAS5 repressed the TGF-β1-induced transformation of pericytes into myofibroblasts

**Fig. 3:**
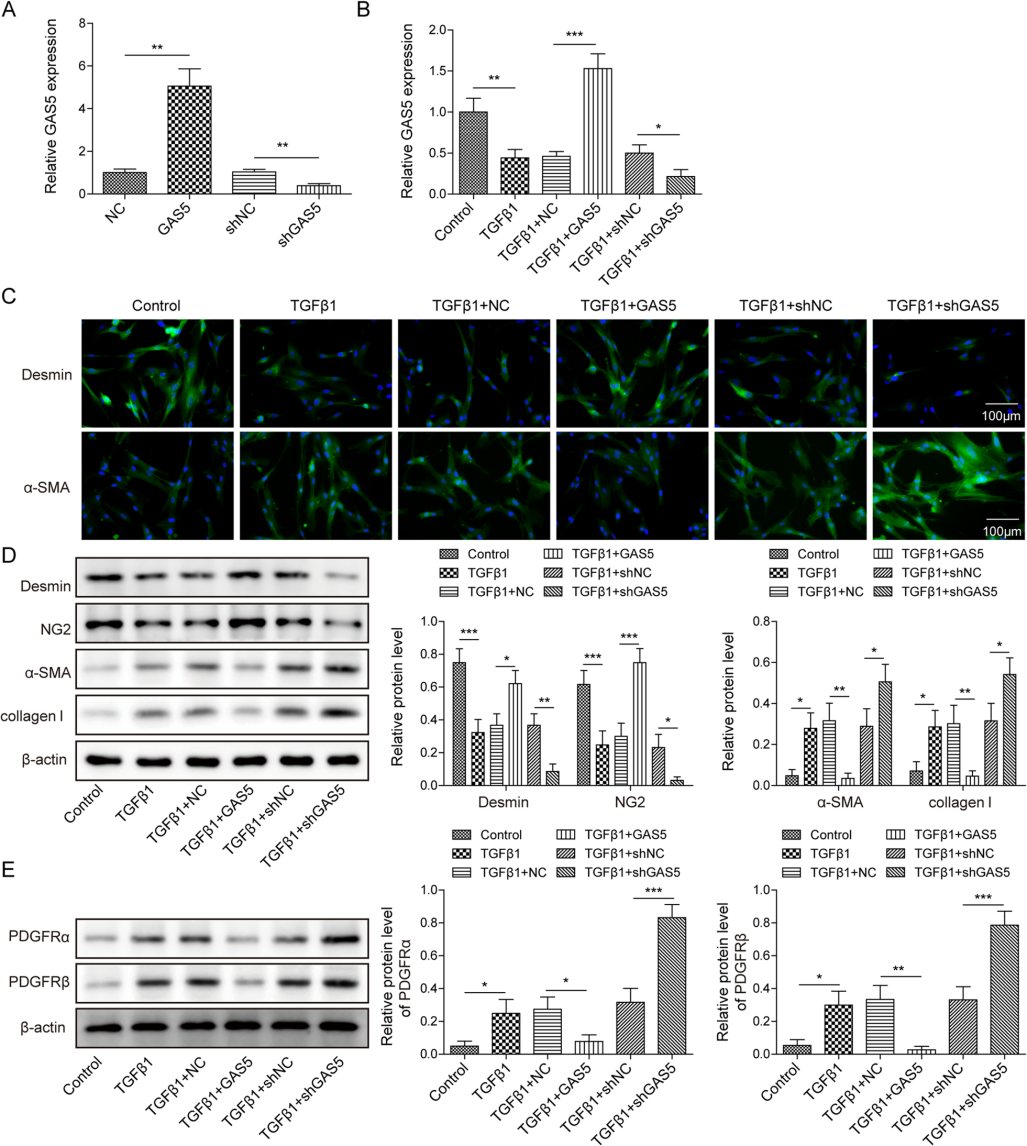
GAS5 repressed the TGF-β1-induced pericyte–myofibroblast transformation. Pericytes were transfected with pcDNA3.0 containing GAS5 (GAS5) or shGAS5 and then exposed to TGF-β1 (10 ng/mL) for 12 h. They were divided into six groups: control, TGF-β1, TGF-β1 + vector, TGF-β1 + GAS5, TGF-β1 + shNC, and TGF-β1 + shGAS5. **A**, **B** RT-qPCR examination of the GAS5 level. **C** Immunofluorescence staining detection of desmin and α-SMA expressions. **D** Western blotting analysis of the protein levels of desmin, NG-2, α-SMA, and collagen I. **E** Western blotting was used to detect the expression of PDGFR α/β. For A–E, n = 3. *P < 0.05, **P < 0.01, ***P < 0.001

Given the differential expression of GAS5 in IPF, we further investigated the impact of GAS5 on TGF-β1-induced pericyte–myofibroblast transformation. The overexpression or silencing efficiency of GAS5 in pericytes was confirmed by RT-qPCR (Fig. 3A, Additional file 1: Fig. S1A, B). The result indicated that shGAS5-1# was adopted in the following experiments. As shown in Fig. 3B, the declined expression of GAS5 in TGF-β1-challenged pericytes was reversed by GAS5 overexpression. Contrarily, GAS5 depletion further reduced the GAS5 level in TGF-β1-stimulated pericytes. In addition, immunofluorescence staining demonstrated that the TGF-β1-induced reduction in desmin expression and increase in α-SMA expression could be counteracted by GAS5 overexpression but reinforced by GAS5 knockdown (Fig. 3C). Furthermore, enforced GAS5 expression reversed the downregulation of desmin and NG2 and upregulation of α-SMA, collagen I, and PDGFR α/β mediated by TGF-β1 exposure, GAS5 depletion yielded opposite results (Fig. 3D, E). Therefore, GAS5 could repress the TGF-β1-induced transformation of pericytes into myofibroblasts.

### PDGFR α/β facilitated pericyte–myofibroblast transformation induced by TGF-β1

**Fig. 4:**
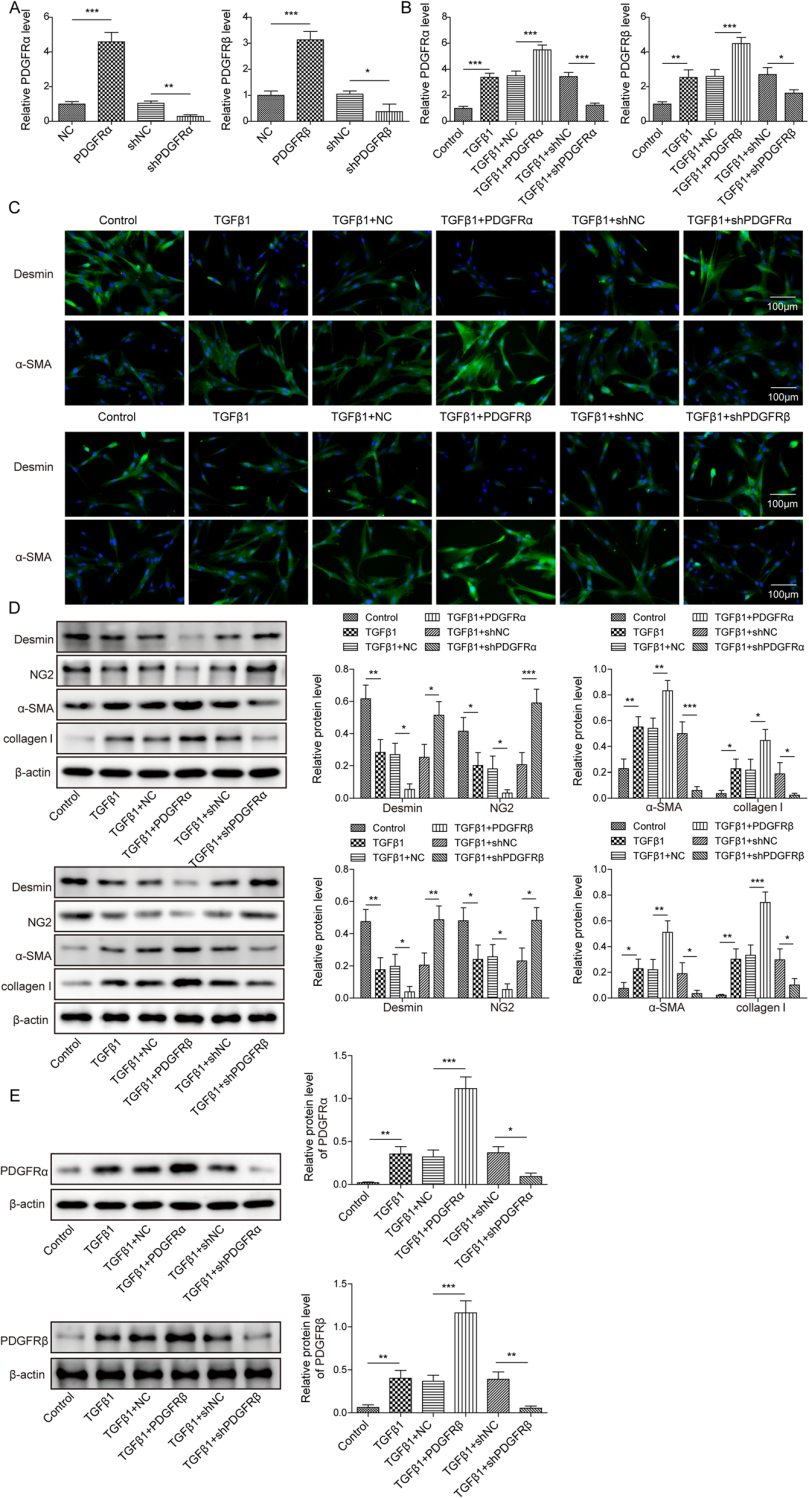
PDGFR α/β facilitated pericyte–myofibroblast transformation induced by TGF-β1. Pericytes were transfected with pcDNA3.0 containing PDGFR α/β (PDGFR α/β) or shPDGFR α/β and then exposed to TGF-β1 (10 ng/mL) for 12 h. They were divided into the following groups: control, TGF-β1, TGF-β1 + vector, TGF-β1 + PDGFR α/β, TGF-β1 + shNC, TGF-β1 + shPDGFR α/β. **A**, **B** The mRNA expression of PDGFR α/β was examined via RT-qPCR. **C** Immunofluorescence staining was employed to evaluate desmin and α-SMA expressions. **D**, **E** Western blotting analysis of the protein levels of desmin, NG-2, α-SMA, collagen I, and PDGFR α/β. For A–E, n = 3. *P < 0.05, **P < 0.01, ***P < 0.001

To explore the function of PDGFR α/β in TGF-β1-induced pericyte–myofibroblast transformation, pericytes were transfected with pcDNA3.0 containing PDGFR α/β or shPDGFR α/β. As confirmed by RT-qPCR and Western blotting, PDGFR α/β was overexpressed or silenced in pericytes following transfection (Fig. 4A, Additional file 1: Fig. S1C–F). shPDGFRα-4# and shPDGFRβ-4# exhibited the highest silencing efficiency, which were selected for future studies (Fig. S1C–F). Furthermore, the TGF-β1-induced increase in the PDGFR α/β levels was enhanced by PDGFR α/β overexpression but abolished by PDGFR α/β knockdown (Fig. 4B). Immunofluorescence examination revealed that the decreased desmin expression and increased α-SMA expression in TGF-β1-exposed pericytes could be strengthened by PDGFR α/β overexpression; however, opposite changes were observed after PDGFR α/β silencing (Fig. 4C). Consistently, Western blotting confirmed that the TGF-β1-induced downregulation of desmin and NG2 and the upregulation of α-SMA, collagen I, and PDGFR α/β. PDGFR α/β were reinforced by PDGFR α/β overexpression but abrogated by PDGFR α/β depletion (Fig. 4D, E). These findings indicated that the TGF-β1-induced transformation of pericytes into myofibroblasts was promoted by PDGFR α/β.

### GAS5 interacted with KDM5B to regulate PDGFR α/β expression

**Fig. 5:**
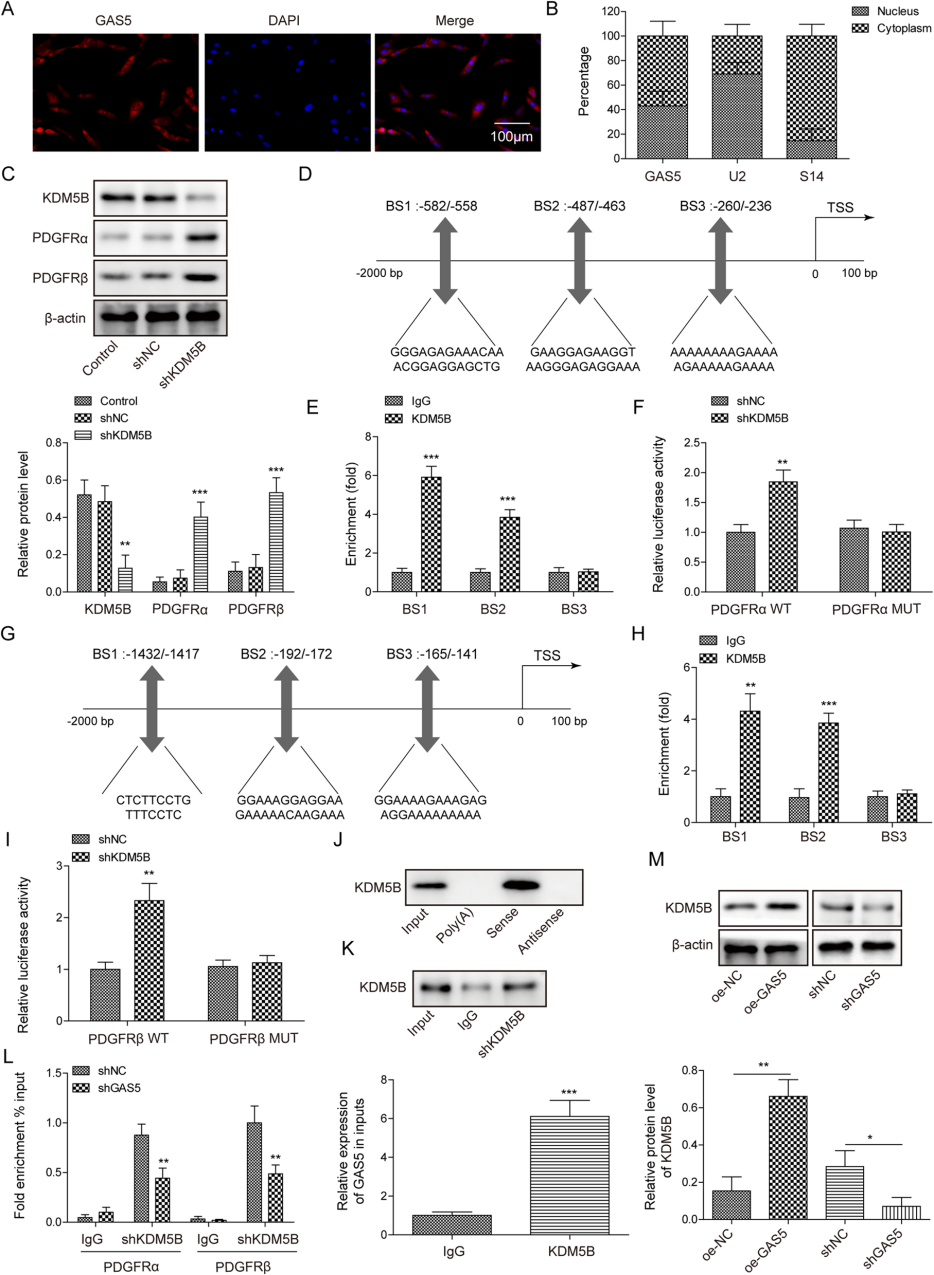
GAS5 recruited KDM5B to repress PDGFR α/β expression. **A** FISH assay was employed to observe the distribution of GAS5 in pericytes. **B** The nuclear and cytoplasmic expression of GAS5 in pericytes was detected via RT-qPCR. **C** Western blotting analysis of the protein levels of KDM5B and PDGFR α/β after KDM5B silencing. **D/G** The AnimalTFDB database predicted the binding sites of KDM5B in the promoter of PDGFR α/β. **E**/**H** ChIP assay was employed to examine the binding between KDM5B and the PDGFR α/β promoter. **F/I** Dual-luciferase assay confirmed the interaction between DKM5B and PDGFR α/β. **J** The interaction between GAS5 and KDM5B was assessed via RNA pull-down assay. **K** The direct binding between GAS5 and KDM5B was confirmed by RIP assay. **L** The binding between KDM5B and the PDGFR α/β promoter after GAS5 knockdown was detected via ChIP assay. **M** The interplay between GAS5 and KDM5B after GAS5 silencing or overexpression was examined via RNA pull-down assay. For **A**–**M** n = 3. *P < 0.05, **P < 0.01, ***P < 0.001

We explored the underlying mechanism of GAS5-mediated modulation of PDGFR α/β expression during IPF progression. The FISH assay revealed that GAS5 was distributed in the cytoplasm and nucleus of pericytes (Fig. 5A). Furthermore, GAS5 was shown to be a little more located in the cytoplasm than in the nucleus (Fig. 5B). The silencing efficiency of shKDM5B-1-4# was confirmed by Western blotting, and shKDM5B-3#, which has the highest silencing efficiency, was selected (Additional file 1: Fig. S1G). Notably, we found that the PDGFR α/β levels were significantly elevated in KDM5B-depleted pericytes (Fig. 5C). The AnimalTFDB database predicted two binding sites (BS1-2) of KDM5B in the PDGFR α/β promoter (Fig. 5D, G). The ChIP assay validated that KDM5B directly bound to BS2 and BS3 in the PDGFR α/β promoter (Fig. 5E, H). In addition, the luciferase activity of WT PDGFR α/β was evidently increased after KDM5B silencing (Fig. 5F, I). As presented in Additional file 2: Fig. S2A, the binding of GAS5 to KDM5B was predicted using the RNA–Protein Interaction Prediction (RPISeq) database (http://pridb.gdcb.iastate.edu/RPISeq/index.html). The possibility of the binding between GAS5 and KDM5B was relatively high (RF = 0.85, SVM = 0.99). The secondary structure of GAS5 is presented in Additional file 2: Fig. S2B. The interaction between GAS5 and KDM5B was also confirmed by RNA pull-down and RIP assays (Fig. 5J, K). Furthermore, GAS5 depletion weakened the binding of KDM5B to the PDGFR α/β promoter (Fig. 5L). Also, the binding between GAS5 and KDM5B was enhanced by GAS5 overexpression but restrained by GAS5 deficiency (Fig. 5M). As presented in Additional file 2: Fig. S2C, RNA pull-down assay detected the binding of a series of GAS5 mutants (GAS5△1, GAS5△2, GAS5△3, and GAS5△4) to KDM5B. The results indicated that KDM5B could bind to GAS5△3 (Additional file 2: Fig. S2C). Meanwhile, the domain patterns of KDM5B binding to GAS5 were predicted using catRAPID (http://service.tartaglialab.com/update_submission/508192/f1d6a86b9b). We found that KDM5B domains JmJN(32-73AA), ARID(97-187AA), and JmjC(453-619AA) might bind to GAS5 (Fig. S2D). Further RNA pull-down assay determined the binding of various KDM5B splice variants (KDM5B△1 [loss of JmJN], KDM5B△2 [loss of ARID], KDM5B△3 [loss of JmjC]) to GAS5. The results indicated that the KDM5B JmjC (453-619AA) domain could directly interact with GAS5 (Additional file 2: Fig. S2D). Overall, PDGFR α/β transcription and expression were modulated by GAS5 through interaction with KDM5B.

### GAS5 promoted H3K4me2/3 demethylation in PDGFRα/β promoter by recruiting KDM5B

**Fig. 6:**
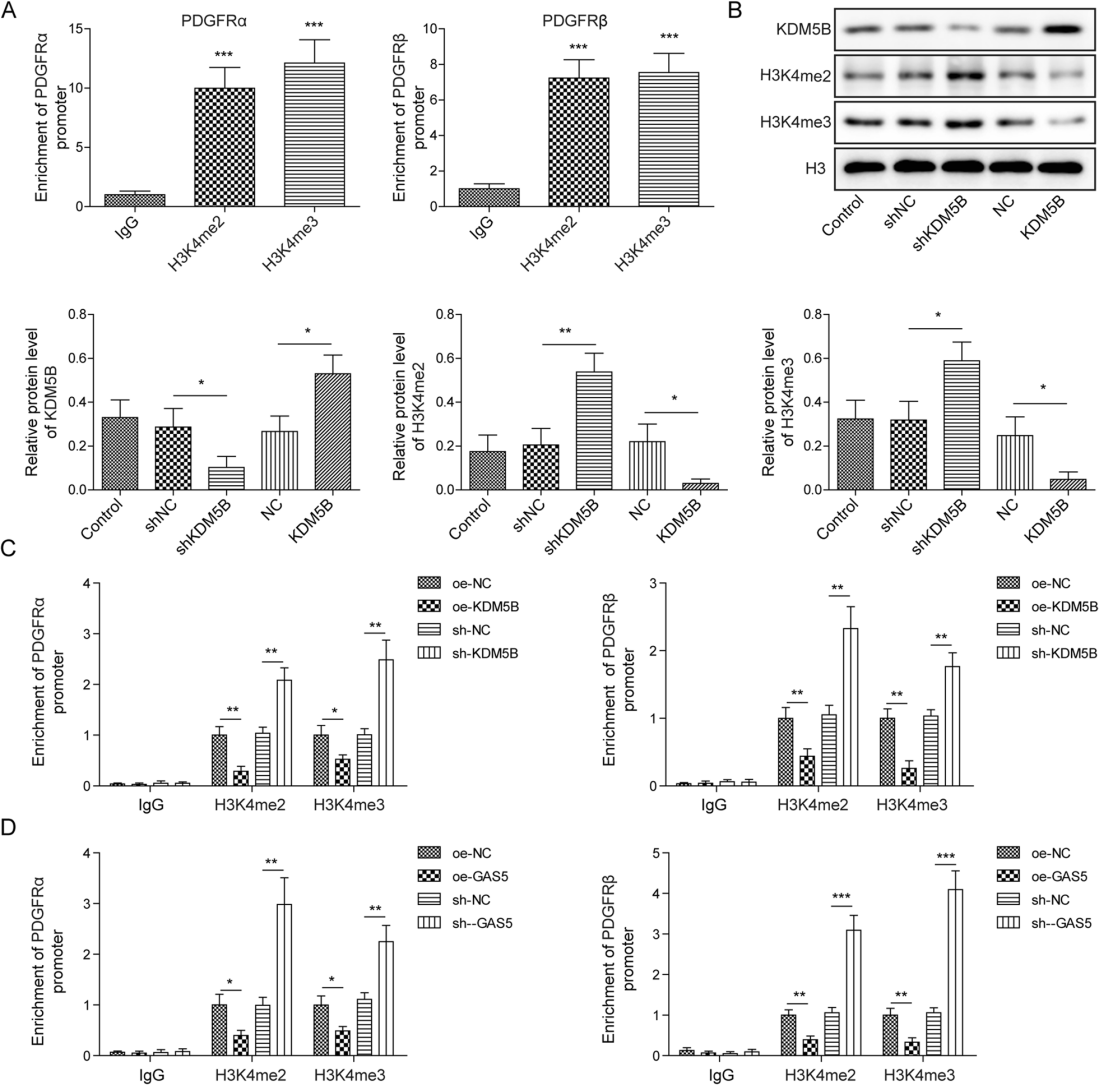
GAS5 facilitated H3K4me2/3 demethylation to downregulate PDGFRα/β by recruiting KDM5B. **A** The binding between H3K4me2/3 and the PDGFR α/β promoter was confirmed by ChIP assay. **B** The protein levels of KDM5B and H3K4me2/3 after KDM5B knockdown or overexpression were detected via Western blotting. **C** ChIP assay analysis of the binding between H3K4me2/3 and the PDGFR α/β promoter in KDM5B-silenced or KDM5B-overexpressed pericytes. **D** The interaction between H3K4me2/3 and the PDGFR α/β promoter after GAS5 silencing or overexpression was determined via ChIP assay. For A–D, n = 3. *P < 0.05, **P < 0.01, ***P < 0.001

To further elucidate the downstream mechanism, H3K4me2/3 attracted our attention. As presented in Fig. 6A, PDGFRα/β enrichment was significantly enhanced after precipitation with the H3K4me2 or H3K4me3 antibody. As presented in Additional file 1: Fig. S1H, the overexpression efficiency of KDM5B was confirmed by Western blotting. Furthermore, KDM5B overexpression upregulated H3Kme2/3 in pericytes, whereas KDM5B deficiency downregulated H3K4me2/3 (Fig. 6B). We also found that the interplay between PDGFRα/β and H3K4me2/3 was repressed by KDM5B silencing but promoted by KDM5B overexpression (Fig. 6C). Similar results indicated that the direct binding between PDGFRα/β and H3K4me2/3 was suppressed in GAS5-overexpressed cells but enhanced in GAS5-depleted cells (Fig. 6D). Collectively, GAS recruited KDM5B to modulate H3K4me2/3 demethylation in the PDGFR α/β promoter.

### GAS5 repressed TGF-β1-induced pericyte–myofibroblast transformation via the KDM5B/PDGFR α/β signaling pathway

**Fig. 7:**
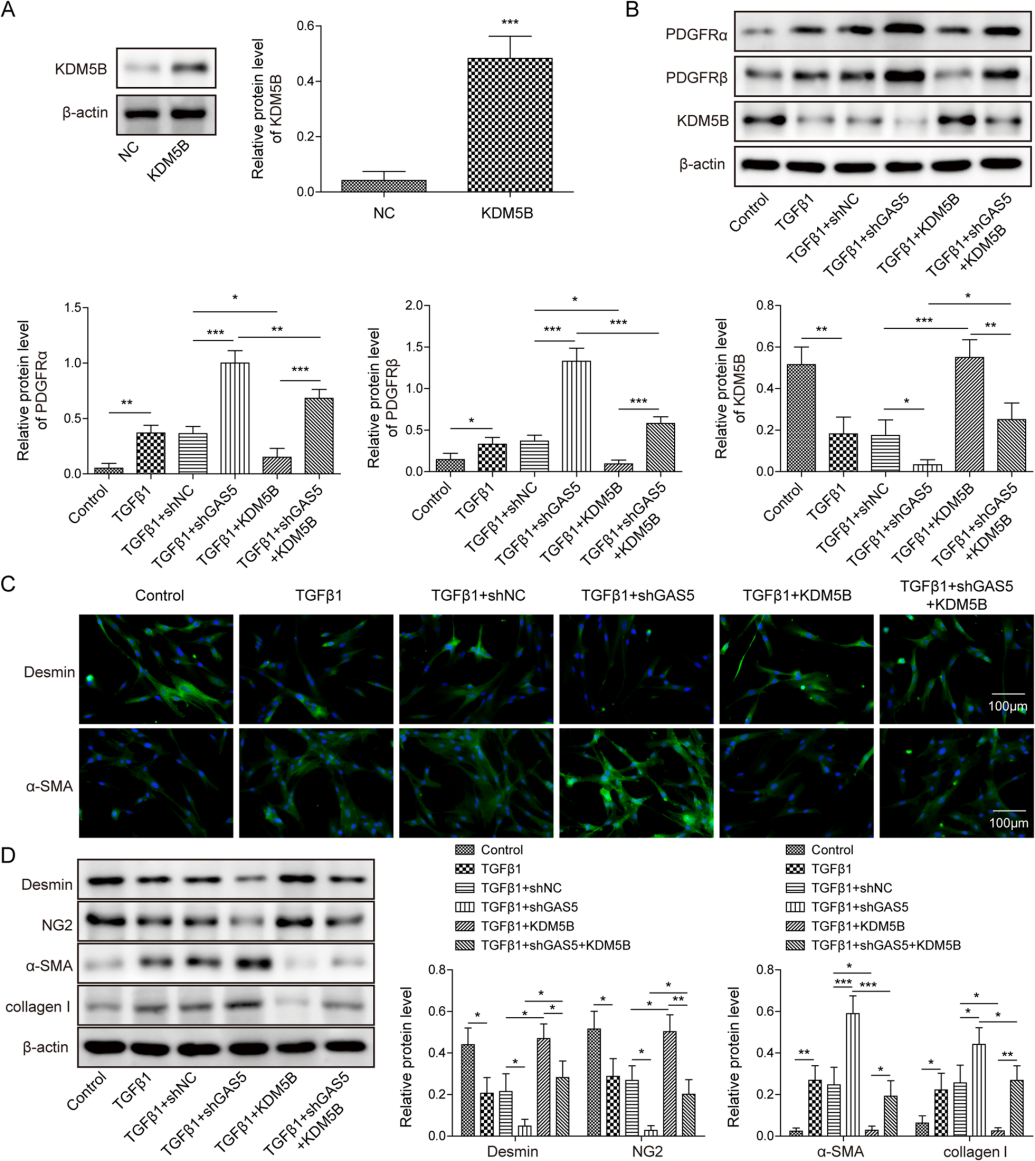
GAS5 repressed TGF-β1-induced pericyte–myofibroblast transformation by regulating the KDM5B/PDGFR α/β signaling pathway. Pericytes were transfected with pcDNA3.0 containing KDM5B (KDM5B) and/or shGAS5 and then exposed to TGF-β1 (10 ng/mL) for 12 h. They were divided into the following groups: control, TGF-β1, TGF-β1 + NC, TGF-β1 + shGAS5, TGF-β1 + KDM5B, and TGF-β1 + shGAS5 + KDM5B. **A**, **B** Western blotting analysis of the expressions of KDM5B and PDGFR α/β after KDM5B overexpression. **C** Immunofluorescence staining to evaluate the expressions of desmin and α-SMA. **D** The protein levels of desmin, NG-2, α-SMA, and collagen I were assessed via Western blotting. For A–D, n = 3. *P < 0.05, **P < 0.01, ***P < 0.001

To determine whether the KDM5B/PDGFRα/β signaling pathway was involved in the GAS5-mediated transformation of pericytes to myofibroblasts, pericytes were transfected with shGAS5, pcDNA3.0-KDM5B, or a combination of them. KDM5B was overexpressed in pericytes transfected with pcDNA3.0-KDM5B (Fig. 7A). As presented in Fig. 7B, the increased PDGFRα/β expression and reduced KDM5B expression in TGF-β1-exposed pericytes were augmented by GAS5 knockdown. However, KDM5B overexpression yielded opposite results and counteracted above shGAS5-mediated changes (Fig. 7B). Furthermore, KDM5B overexpression increased desmin and NG2 expressions and reduced α-SMA and collagen I expressions in TGF-β1-stimulated pericytes and abolished GAS5 depletion-induced reduction in desmin and NG2 expressions and elevation in α-SMA and collagen I expressions (Fig. 7C, D). These data indicated that the KDM5B/PDGFR α/β axis participated in the GAS5-mediated inhibition of pericyte–myofibroblast transformation.

### GAS5 inhibited PF progression in bleomycin-induced mice in vivo

**Fig. 8:**
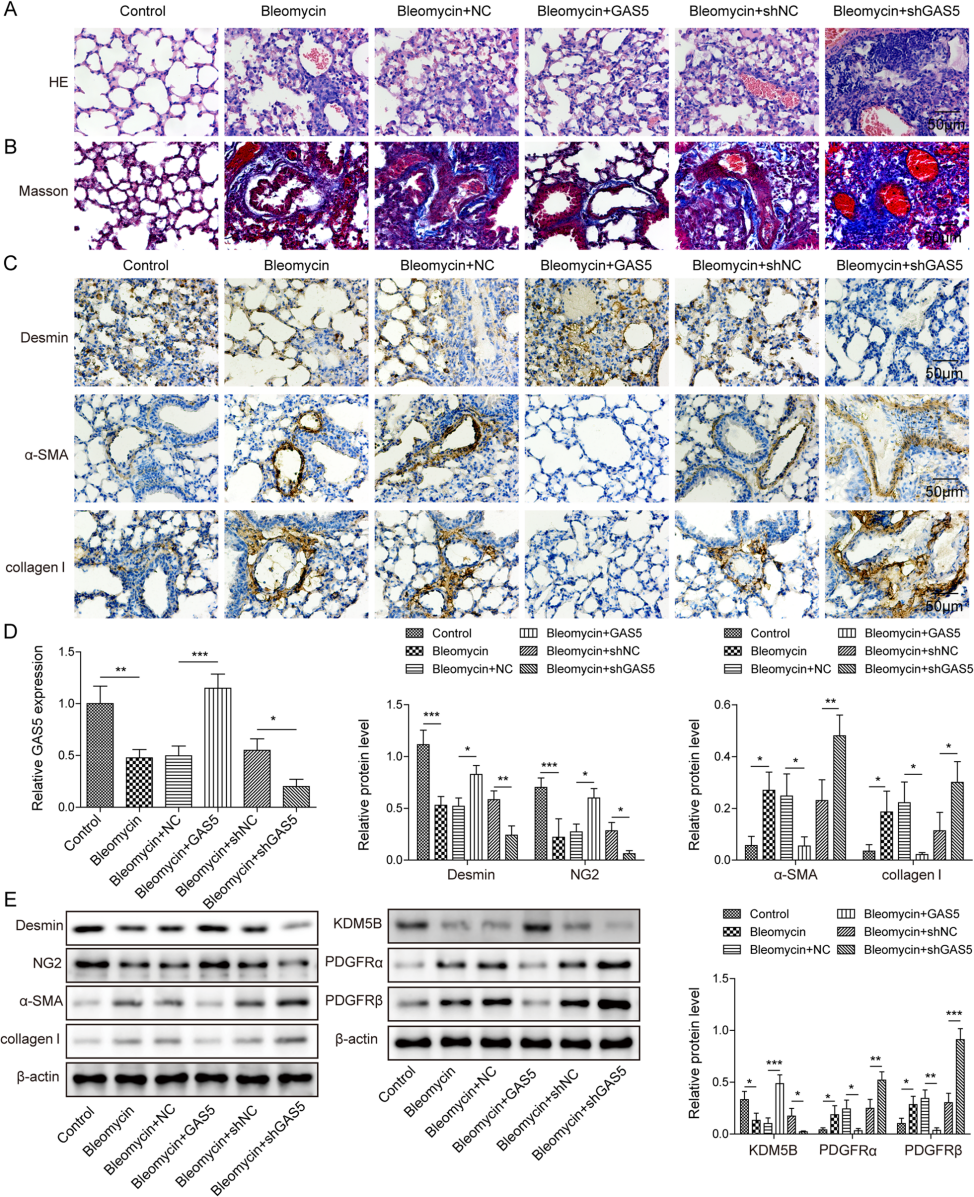
GAS5 inhibited pulmonary fibrosis in the mouse model of pulmonary fibrosis in vivo. There were six experimental groups: control, bleomycin, bleomycin + NC, bleomycin + GAS5, bleomycin + shNC, and bleomycin + shGAS5. **A**, **B** The degree of pulmonary fibrosis and collagen deposition in the lungs of mice was assessed via H&E and Masson staining. **C** The expressions of desmin, α-SMA, and collagen I in the lung tissues were determined via immunohistochemical staining. **D** RT-qPCR analysis of GAS5 expression in different groups. **E** The protein levels of desmin, NG-2, α-SMA, collagen I, KDM5B, and PDGFR α/β in the lungs were examined via Western blotting. For A–E, n = 6. *P < 0.05, **P < 0.01, ***P < 0.001

Finally, we validated the function of GAS5 in the mouse model of PF in vivo. Histopathological examination revealed that the pathological changes and PF that occurred in the bleomycin group were attenuated by GAS5 overexpression but aggravated by GAS5 knockdown (Fig. 8A). Furthermore, GAS5 overexpression attenuated collagen deposition in the lungs of PF mice, whereas GAS5 deficiency yielded opposite results (Fig. 8B). Immunohistochemical staining showed that the bleomycin-induced reduction in desmin expression and increase in α-SMA and collagen I expressions were attenuated in the GAS5 overexpression group but aggravated in the shGAS5 group (Fig. 8C). RT-qPCR confirmed the downregulation of GAS5 in the bleomycin group and overexpression or silencing of GAS5 in the PF mouse model (Fig. 8D). Accordingly, GAS5 overexpression increased the low levels of desmin, NG-2, and KDM5B but decreased the high levels of α-SMA, collagen I, and PDGFRα/β in the lung tissues of PF mice. Conversely, the reverse effect of GAS5 knockdown was observed (Fig. 8E). The above results indicated that GAS5 attenuated lung fibrosis in bleomycin-induced mice in vivo by regulating the KDM5B/PDGFR α/β signaling pathway.

## Discussion

To date, IPF remains a progressive and fatal disorder, with increasing incidence and prevalence rates (Saito et al. 2019). The median survival of IPF is 2–3 years after diagnosis (Sharif 2017). It has a high mortality rate, and the available treatments for this disease are few; thus, safe and effective therapeutic agents need to be developed. Growing evidence has indicated that pericytes are the origin of myofibroblasts that contribute to the incidence of IPF (Yamaguchi et al. 2020); thus, elucidation of the mechanism underlying pericyte–myofibroblast transformation is crucial for the development of novel treatment for IPF. This study found that GAS5 was downregulated whereas PDGFR α/β was upregulated in the lungs of patients and mice with IPF. Moreover, GAS5 overexpression or PDGFR α/β knockdown inhibited TGF-β1-induced pericyte–myofibroblast differentiation. GAS5 recruited KDM5B to repress PDGFR α/β expression via H3K4me2/3 demethylation. Finally, GAS5 overexpression attenuated lung fibrosis in bleomycin-induced mice by regulating the KDM5B/PDGFR α/β signaling pathway. Our observations indicated that GAS5 is a novel target for the treatment of IPF.

LncRNAs have been found to participate in IPF progression. For example, Yang et al. found that lncRNA ZFAS1 triggered ferroptosis to promote IPF progression via the miR-150-5p/SLC38A1 signaling pathway (Yang et al. 2020). The influence of GAS5 on fibrosis has been widely documented in multiple tissues. For instance, GAS5 was reported to attenuate renal interstitial fibrosis in diabetic rats by regulating the EZH2/MMP9 signaling pathway (Zhang et al. 2020). A previous study demonstrated that GAS5 attenuated renal fibrosis by modulating the Smad3/miRNA-142-5p axis (Zhang et al. 2021). Tao et al. showed that GAS5 suppression by MeCP2 led to cardiac fibrosis (Tao et al. 2020). To date, the function of GAS5 in IPF has not yet been elucidated. The mouse model of intratracheal bleomycin administration is the most well-characterized animal model currently available for preclinical PF research (Jenkins et al. 2017). Thus, we used bleomycin to create an in vivo PF mouse model in this study. Our data indicated that GAS5 was downregulated in the lungs of patients and mice with IPF. Furthermore, we observed an enhanced expression of PDGFRα/β in the lungs of these patients. Excessive activation of the PDGFR α/β signaling pathway may result in tissue fibrosis (Ieronimakis et al. 2016). It has also been suggested that inactivation of the PDGFRα/β signaling pathway is essential for delaying IPF progression (Kishi et al. 2018; Vuorinen et al. 2007). Fibrosis progression caused by myofibroblast activation may destroy the normal structure and function of the lung tissue. Inhibiting myofibroblast activation is an effective strategy for treating fibrosis (Morelli et al. 2019; Feng et al. 2022). Wang et al. demonstrated that inhibition of exosomal microRNA-92a restrained the activation of cardiac fibroblasts, thereby attenuating cardiac fibrosis (Wang et al. 2020). TGF-β1, a critical inducer of tissue fibrosis (including IPF), can activate myofibroblasts and induce excessive ECM deposition (Hu et al. 2018; Zainal Abidin et al. 2021; Jordan et al. 2021). Inactivation of the TGFβ signaling pathway was proven to reduce the level of fibrosis-related proteins in myofibroblasts (Yousefi et al. 2021). α-SMA is a common marker for myofibroblasts, and ECM deposition is predominantly indicated by increased collagen I expression (Li et al. 2019). Herein, our findings indicated that GAS5 overexpression or PDGFRα/β deficiency effectively inhibited TGF-β1-induced pericyte–myofibroblast transformation by repressing α-SMA and collagen I expressions and elevating pericyte markers desmin and NG2 expression. Conversely, GAS5 silencing or PDGFRα/β overexpression yielded opposite results. Collectively, loss of GAS5 or PDGFRα/β overexpression promoted IPF progression by inducing pericyte–myofibroblast transformation.

In view of the important function of GAS5 and PDGFRα/β in IPF, we further elucidated the underlying regulatory mechanism. LncRNAs have been demonstrated to act as scaffolds, decoys, or miRNA sequesters, thereby exerting their molecular functions (Wang and Chang 2011). GAS5 has been shown to affect various signaling cascades by recruiting protein for the formation of RNA–protein complexes. For instance, it has been suggested that GAS5 inactivates the TGF-β/Smad signaling pathway during skin fibrosis by facilitating PPM1A-mediated Smad3 dephosphorylation (Tang et al. 2020). Interestingly, bioinformatics analysis revealed that both GAS5 and PDGFRα/β bind to KDM5B. KDM5B, as a histone demethylase, plays pivotal roles in H3K4me2/3 demethylation (Zhang et al. 2014). H3K4me2/3 residue has been recognized as a gene transcription initiation site, and H3K4me2/3 demethylation indicates the inhibition of transcription, suggesting its active role in suppressing gene expression (Zheng et al. 2019). A previous study demonstrated that KDM5B participated in osteoblast differentiation regulation by epigenetic modulation of Runx2 gene via H3K4me3 (Rojas et al. 2015). Here, we found that GAS5 acted as a scaffold that recruited KDM5B to the PDGFRα/β promoter and consequently repressed PDGFRα/β expression via H3K4me2/3 demethylation. Therefore, GAS5 inhibited pericyte–myofibroblast transformation by recruiting KDM5B to repress PDGFRα/β expression.

## Conclusion

**Fig. 9:**
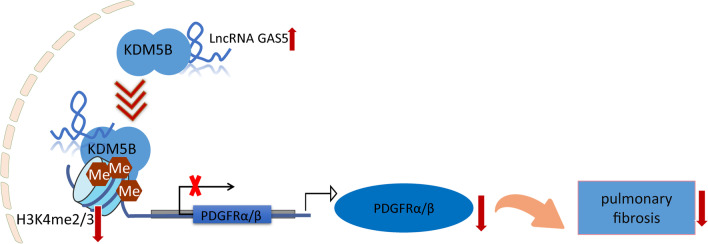
A schematic map illustrating the role and mechanism of lncRNA GAS5 in pulmonary fibrosis. GAS5 recruited KDM5B to facilitate H3K4me2/3 demethylation that reduced PDGFR α/β transcription and expression, thus inhibiting pulmonary fibrosis

In conclusion, the current study demonstrated that GAS5 overexpression restrained TGF-β1-induced pericyte–myofibroblast transformation and bleomycin-induced PF by reducing PDGFR α/β expression through interaction with KDM5B to promote H3K4me2/3 demethylation, suggesting a negative regulatory role of GAS5 in the etiology of IPF (Fig. 9). Our findings identified GAS5 as a potential therapeutic target for IPF.

## Supplementary Information

**Additional file 1: Figure S1. **Confirmation of the silencing or overexpression efficiency.** (A)** RT-qPCR analysis of the expression of GAS5 in pericytes after transfection with shGAS5-1–4#. **(B)** RT-qPCR analysis of the level of GAS5 in pericytes after transfection with pcDNA3.0-GAS5. **(C and D)** Western blotting analysis of the levels of PDGFRα/β in pericytes transfected with shPDGFRα/β-1-4#. **(E and F)** The protein levels of PDGFRα/β in pericytes after transfection with pcDNA3.0-PDGFR α/β were evaluated via Western blotting. **(G)** Western blotting analysis of the level of KDM5B in pericytes transfected with shKDM5B-1-4#. **(H)** The protein level of KDM5B in pericytes after transfection with pcDNA3.0-KDM5B was detected via Western blotting. *P < 0.05, **P < 0.01, ***P < 0.001.

**Additional file 2: Fig. S2. **Direct interaction between GAS5 and KDM5B.** (A)** The RNA–Protein Interaction Prediction (RPISeq) database predicted the binding sites between GAS5 and KDM5B. **(B)** Secondary structure of GAS5. **(C)** The binding of a series of GAS5 mutants (GAS5△1, GAS5△2, GAS5△3, and GAS5△4) to KDM5B was validated via RNA pull-down assay. **(D)** RNA pull-down assay analysis of the binding of various KDM5B splice variants (KDM5B△1(loss of JmJN), KDM5B△2(loss of ARID), and KDM5B△3(loss of JmjC)) to GAS5.

## Data Availability

The datasets generated during and/or analysed during the current study are available from the corresponding author on reasonable request.
