# Supplementary figures and images for: LncRNA GAS5 suppresses TGF-β1-induced transformation of pulmonary pericytes into myofibroblasts by recruiting KDM5B and promoting H3K4me2/3 demethylation of the PDGFRα/β promoter
Source: Mol Med. 2023 Mar 14;29:32. doi: 10.1186/s10020-023-00620-x (PMC10015786; doi:10.1186/s10020-023-00620-x)

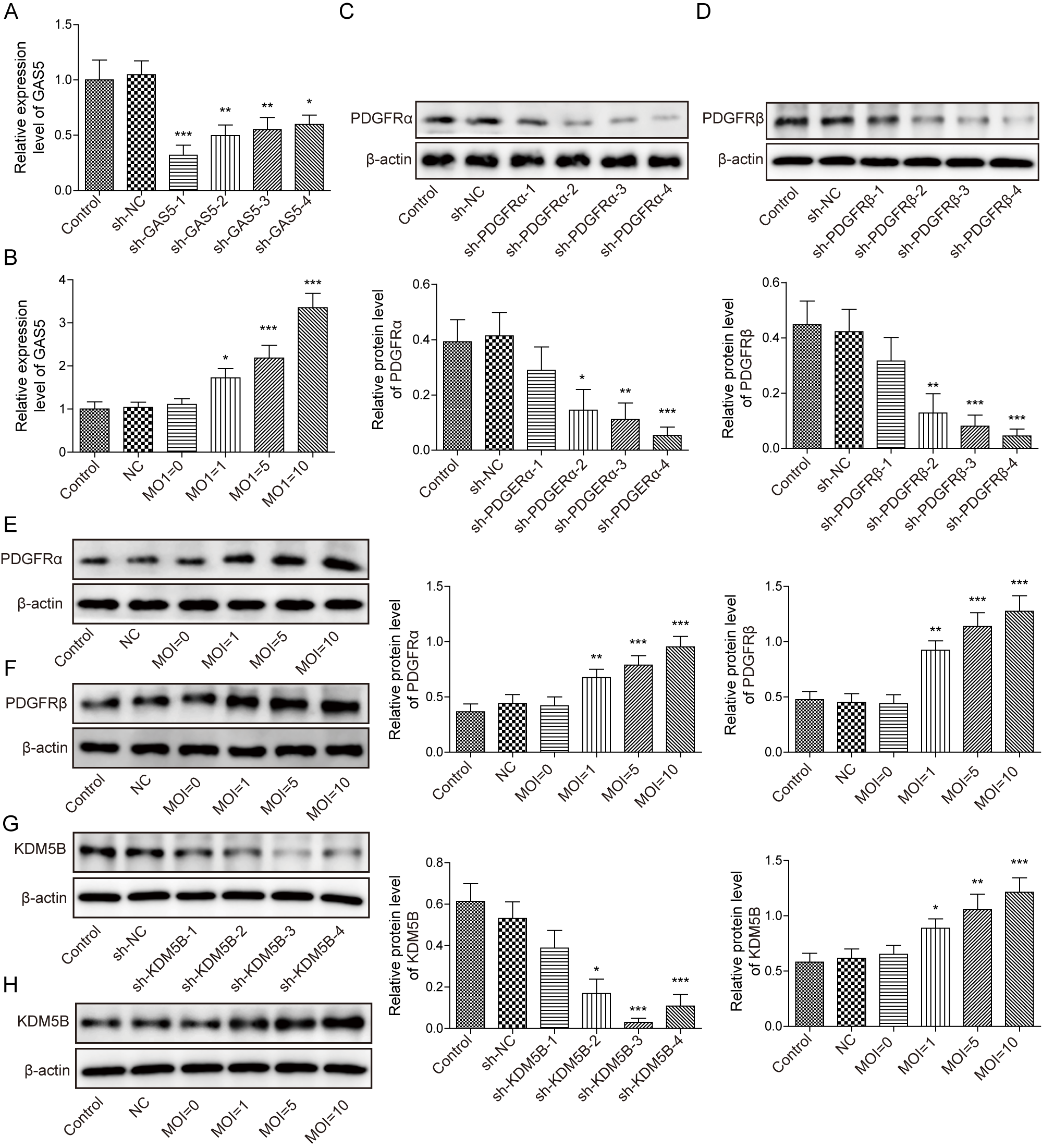

Supplement: Supplementary file 1 — Additional file 1: Figure S1. Confirmation of the silencing or overexpression efficiency. (A) RT-qPCR analysis of the expression of GAS5 in pericytes after transfection with shGAS5-1–4#. (B) RT-qPCR analysis of the level of GAS5 in pericytes after transfection with pcDNA3.0-GAS5. (C and D) Western blotting analysis of the levels of PDGFRα/β in pericytes transfected with shPDGFRα/β-1-4#. (E and F) The protein levels of PDGFRα/β in pericytes after transfection with pcDNA3.0-PDGFR α/β were evaluated via Western blotting. (G) Western blotting analysis of the level of KDM5B in pericytes transfected with shKDM5B-1-4#. (H) The protein level of KDM5B in pericytes after transfection with pcDNA3.0-KDM5B was detected via Western blotting. *P < 0.05, **P < 0.01, ***P < 0.001. [file 10020_2023_620_MOESM1_ESM.tif]

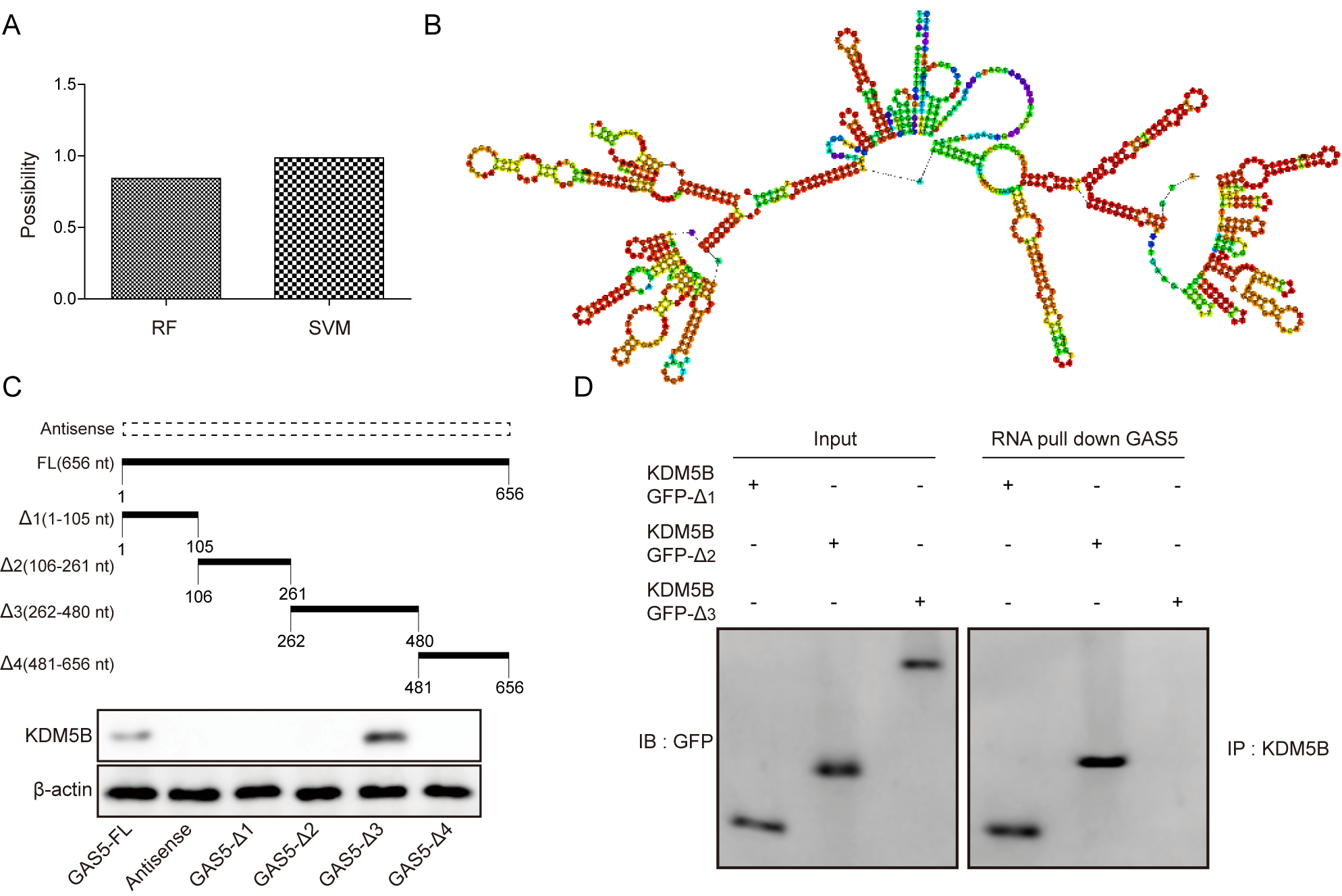

Supplement: Supplementary file 2 — Additional file 2: Fig. S2. Direct interaction between GAS5 and KDM5B. (A) The RNA–Protein Interaction Prediction (RPISeq) database predicted the binding sites between GAS5 and KDM5B. (B) Secondary structure of GAS5. (C) The binding of a series of GAS5 mutants (GAS5△1, GAS5△2, GAS5△3, and GAS5△4) to KDM5B was validated via RNA pull-down assay. (D) RNA pull-down assay analysis of the binding of various KDM5B splice variants (KDM5B△1(loss of JmJN), KDM5B△2(loss of ARID), and KDM5B△3(loss of JmjC)) to GAS5. [file 10020_2023_620_MOESM2_ESM.tif]
